# Association of Dioxin and Other Persistent Organic Pollutants (POPs) with Diabetes: Epidemiological Evidence and New Mechanisms of Beta Cell Dysfunction

**DOI:** 10.3390/ijms15057787

**Published:** 2014-05-05

**Authors:** Vincenzo De Tata

**Affiliations:** Department of Translational Research and New Technologies in Medicine and Surgery, University of Pisa, Via Roma, 55, Scuola Medica, 56126 Pisa, Italy; E-Mail: vincenzo.detata@med.unipi.it; Tel.: +39-050-2218-546; Fax: +39-050-2218-577

**Keywords:** dioxin, diabetes, endocrine disruptors, persistent organic pollutants, beta cells, insulin secretion

## Abstract

The worldwide explosion of the rates of diabetes and other metabolic diseases in the last few decades cannot be fully explained only by changes in the prevalence of classical lifestyle-related risk factors, such as physical inactivity and poor diet. For this reason, it has been recently proposed that other “nontraditional” risk factors could contribute to the diabetes epidemics. In particular, an increasing number of reports indicate that chronic exposure to and accumulation of a low concentration of environmental pollutants (especially the so-called persistent organic pollutants (POPs)) within the body might be associated with diabetogenesis. In this review, the epidemiological evidence suggesting a relationship between dioxin and other POPs exposure and diabetes incidence will be summarized, and some recent developments on the possible underlying mechanisms, with particular reference to dioxin, will be presented and discussed.

## 1. Introduction

Diabetes mellitus is one of the most common chronic diseases, and its prevalence has exploded over the last several decades. The World Health Organization and the International Diabetes Federation estimated that the prevalence of diabetes increased from 100 to 135 million affected adults worldwide in 1994–1995 to approximately 336 million in 2011, and it is expected to rise to 439 million by 2030 [1–3]. Since these changes occurred too rapidly to simply reflect genetic causes and rates vary widely by country, the vast majority of new cases of diabetes are likely to be caused by changes in lifestyle and/or environment during the last few decades. Therefore, the possibility that a corresponding distribution of toxic substances might account for the geographic variations in diabetes prevalence was initially suggested in 1972 [4]. In this regard, it might be worthy to mention that in the U.S., diabetes rates have increased in concordance with the national production of synthetic organic chemicals [5]. Dioxins and other persistent organic pollutants (POPs) (such as polychlorinated biphenyls, dichlorodiphenyldichloroethylene (DDE), the main degradation product of the pesticide, dichlorodiphenyltrichloroethane (DTT), trans-nonachloro, hexachlorobenzene and the hexachlorocyclohexanes) are generally considered to be the most likely candidates as potential risk factors for type 2 diabetes [6], since they may act as endocrine disruptors. Endocrine disrupting chemicals have been defined by the Environmental Protection Agency (EPA) as “exogenous agents that interfere with the production, release, transport, metabolism, binding, action, or elimination of natural hormones in the body responsible for the maintenance of homeostasis, reproduction, development, and/or behavior” [7–10]. Many of these pollutants (although not all; see, e.g., the case of bisphenol A) were banned in most Western countries in the 1970s and 1980s, with a consequent diminution of their levels in the environment, but they are still detected in humans, especially in those subgroups of the general population that still show an elevated body burden due to dietary habits and current and past exposure [11–13].

## 2. Epidemiological Evidence

Several epidemiological studies contributed to establishing a clear relationship between dioxin (as well as other POPs) exposure and type 2 diabetes incidence (for recent reviews, see [14,15]. This association was firstly reported for occupationally exposed populations and subsequently investigated in the general population.

Many veterans of the Vietnam War were exposed to 2,3,7,8-tetrachlorodibenzo-*p*-dioxin (TCDD or simply dioxin), which was a contaminant in the herbicide formulation, Agent Orange, widely used as a defoliant by the U.S. Air Force from 1961 to 1971 [16]. Even after many years, significant residues of dioxin have been found in their serum [17,18], because of the long half-life of dioxin, which is estimated to be 7.6 years [19]. The follow-up of veterans to assess the possible long-term health consequences of exposure to Agent Orange identified type 2 diabetes as one of the diseases most consistently statistically associated with dioxin exposure [20–24]. Similar conclusions were reached by epidemiological studies carried out on the population exposed to high levels of dioxin as a result of an industrial accident on 10 July 1976, in the trichlorophenol production department of a chemical plant located near the town of Seveso, 25 km north of Milan, Italy [25–27]. A 25-year follow up of the exposed population showed a significantly increased mortality from diabetes mellitus among females [28,29]. This last observation could be of particular relevance in relation to the recently reported sex-specific metabolic disorders induced by a diet containing low doses of TCDD and other POPs in the progeny of obese mice [30]. Other epidemiological studies, with comparable results, were performed in professionally exposed industrial workers [31], Korean Vietnam veterans, who were also exposed to Agent Orange [32], and individuals living near municipal waste incinerators [33] or hazardous waste sites [34]. Similar results were obtained in a 24-year follow-up study of 1054 Yucheng “oil-disease” victims that, during the late 70s, were accidentally exposed to rice-bran oil laced with polychlorinated biphenyls [35]. In contrast, negative results were obtained in professionally exposed workers by other authors [36–41]. In particular, Kerger *et al.* [42], recently re-analyzing the Ranch Hand study, supported the hypothesis that diabetes progression could lead to higher dioxin levels rather than the opposite. In more recent years, the exploration of the epidemiological relationship between dioxin (as well as other correlated POPs) exposure and diabetes incidence has been extended from professionally or accidentally exposed individuals to the general population. In this regard, a preliminary note of caution is required. A main feature of POPs is the fact that these compounds are always present as chemical mixtures, and therefore, when studies involving general populations with only background exposure to POPs are evaluated, data obtained for a single compound cannot be simply attributed to the exclusive effect of that compound, but must rather be viewed as the result of the overall effect of the mixture [43]. Fierens *et al.* [44], in a population-based study in Belgium, firstly found that diabetic patients had significantly increased serum levels of dioxins and coplanar polychlorinated biphenyls. In 2006, using cross-sectional data from the 1999–2002 U.S. National Health and Examination Survey, Lee *et al.* [45,46] reported a strong correlation between serum concentration of POPs (especially organochlorine compounds) and diabetes. The association with serum levels of POPs was subsequently extended by the same group to insulin resistance [47], to the prevalence of metabolic syndrome [48] and to abdominal obesity [49] among non-diabetic adults. These results were further supported by a study of Everett *et al.* [50] showing a significant positive correlation between diabetes and polychlorinated dibenzo-*p*-dioxin, a polychlorinated biphenyl, and DDT in a group of 1830 subjects from the 1999–2002 National Health and Examination Survey. Intriguingly, Lee and co-workers [51,52] hypothesized that serum gamma-glutamyltransferase (GGT) may predict diabetes as a general marker of exposure to various environmental xenobiotics, including diabetes-related POPs. Other studies on general populations have further confirmed the association between POPs exposure and diabetes incidence [53–60].

POPs are generally lipophilic, resistant to degradation and tend to bioaccumulate within the food chain. Therefore, in spite of the general decline of their environmental levels over recent decades, today, they are especially found as contaminants in food. For this reason, some recent studies on the relationship between the consumption of foods, such as fish and marine mammals, and diabetes incidence could be particularly interesting (for a review, see [61]). Jǿrgensen *et al.* [62] showed a significant association between plasma POP levels and impaired beta cell function among Greenland Inuit, while Sharp [63] reported that diabetes prevalence rates are 3–5 times higher among Canadian aboriginals than in the general population, and Philibert *et al.* [64] found a positive correlation between diabetes and serum levels of *p*,*p*′-DDE and some polychlorinated biphenyl congeners in a First Nation community. Finally, Rylander *et al.* [65] reported a significant correlation between plasma POP levels and type 2 diabetes in Swedish fisherman and their wives, and Turyk *et al.* [66] found a similarly significant association in a group of Great Lakes sport fish consumers.

A critical evaluation of the epidemiological literature, which is far from being homogeneous and still presents several gaps and inconsistences that can seriously limit the validity of some of the data reported, is beyond the scope of this review and has been recently addressed elsewhere [14], reaching the main conclusion that POPs have generated particularly strong evidence as risk factors for diabetes in humans [14,43]. New and original ways to study the association between dioxin and other POP exposure with diabetes have been recently proposed. Fujiyoshi *et al.* [67], in order to offer some clues about the mechanism of the diabetogenic action of dioxin in humans, performed a molecular epidemiological investigation in adipose tissue samples from U.S. Air Force Vietnam veterans who were or were not exposed to dioxin. They conducted quantitative reverse-transcribed polymerase chain reaction studies on selected marker mRNAs from these samples and found that the most sensitive and reliable molecular indicator of dioxin-induced diabetes was the ratio of mRNA of glucose transporter 4 (GLUT4) and NFkB, a marker of inflammation. Interestingly, this ratio showed significant correlations to serum dioxin levels and to fasting glucose, not only among the exposed veterans, but even in the comparison group, who have low levels of dioxin comparable to the general public. More recently, Patel *et al.* [68] proposed a model environmental-wide association study (EWAS) to search for environmental factors associated with type 2 diabetes, in which epidemiological data were comprehensively and systematically interpreted in a manner analogous to genome wide association studies (GWASs).

## 3. Mechanisms of Dioxin-Induced Beta Cell Dysfunction

We have seen so far that overall epidemiological evidence supports a positive association between dioxin and other organochlorine POPs exposure and diabetes incidence. However, association does not necessarily imply a causal link, although several biologically plausible explanations have been proposed [6]. For this reason, very recently, a panel of experts that evaluated the association between POPs and diabetes in epidemiological studies in a workshop sponsored by the National Institute of Environmental Health Sciences (NIEHS)/National Toxicology Program (NTP), the U.S. Environmental Protection Agency (EPA) and the Food and Drug Administration National Center for Toxicological Research indicated the need of additional animal and *in vitro* mechanistic studies to clarify the role of POPs in metabolic disease development [14]. Regrettably, the investigation of the biological mechanisms underlying the relationship between dioxin exposure and type 2 diabetes has been rather neglected until recently.

### 3.1. In Vivo Effects of Dioxin Administration

Initially, research on dioxin toxicity was mainly focused on its acutely lethal effects [69–71] in highly sensitive rodents, such as guinea pigs. Subsequently, the realization that human beings are less susceptible to these acute effects shifted the attention on low level chronic effects, such as carcinogenicity [72,73] and reproductive toxicology [74–76], that can be induced in animal models at concentrations close to human background exposure [12]. Among the most common symptoms seen in animal species treated with 2,3,7,8-tetrachlorodibenzo-*p*-dioxin (TCDD, the most toxic of the dioxin-type chemicals and, thus, the most widely used in animal toxicological studies), there is body weight loss or reduced weight gain, mostly related to the loss of fat and muscle tissue (“wasting syndrome”) [77–80]. Adipose tissue loss is associated with hypophagia [81], hyperlipidemia, particularly hypertriglyceridemia [82], probably linked to the TCDD-induced drastic decline in adipose lipoprotein lipase activity [83,84], and with changes in the expression levels of the adipogenesis- and lipogenesis-related proteins in the liver and adipose tissue [85–88] and hypoinsulinemia [89]. The insulin resistance-like symptoms observed in wasting syndrome, such as the loss of body and adipose tissue weight, have been recently attributed to the TCDD-induced activation of TNF-α [90–92] and COX-2 [93]. Since anorexia is a well-known component of TCDD-induced wasting syndrome, the effects of dioxin administration on hypothalamic regions have been also investigated, and several alterations in the pathways regulating body weight and food intake have been reported [79,80,94–98]. On the basis of these observations, dioxin seems to hinder the utilization of the available nutritional elements (e.g., glucose, triglycerides, cholesterol, *etc.*) in the affected animals. Among the metabolic derangements induced by TCDD, those related to glucose homeostasis could play a crucial role in the induction of wasting syndrome. One of the most characteristic alterations induced by TCDD is a marked decrease in glucose uptake by adipose tissue, liver and pancreas *in vivo*, as well as *in vitro* [99–103], mainly caused by the TCDD-induced decline in the titer of glucose transporters [90,104–109]. In particular, it has been proposed that the reduced glucose uptake caused by the decreased GLUT2 expression in beta cells could explain the TCDD-induced lowering of insulin production and secretion by the pancreas [37,110]. Indeed, TCDD can cause hypoinsulinemia both in the rabbit [89] and in the rat [111,112]. More recently, we showed that, in the rat, a single low dose of TCDD (1 μg/kg b.w., well below the usually lethal one, estimated to be around 125 μg/kg), was able to rapidly reduce both pancreatic insulin content and glucose-stimulated insulin secretion from isolated pancreatic islets, indicating an early involvement of the endocrine pancreas in TCDD toxicity [113]. Similar results were also reported by Kurita *et al.* [114]. We explored also the secretory response of isolated islets to the non-glucose secretagogue, 2-ketoisocaproate (2-KIC), which is one of the few physiological substrates apart from glucose that initiates a sustained insulin release from beta cells [115–117], and we found that 2-KIC-induced insulin release was better preserved in TCDD-treated rats, indirectly confirming that the impairment in the secretory performance observed in pancreatic islets isolated from TCDD-treated rats was strictly linked to the failure of glucose to induce insulin secretion in beta cells [113]. In pancreatic beta cells, glucose utilization is regulated by glucokinase, a glucose-specific high-K_m_ enzyme that, at glucose concentrations >5.5 mM, is the rate limiting step for glucose metabolism and, therefore, for glucose-induced insulin release [118,119]. Under these conditions, glucose transport, whose capacity largely exceeds glucose phosphorylation and metabolism, does not play a major role in determining the secretory response [120]. However, when the expression or function of the glucose transporter is significantly reduced, it may limit the access of glucose to glucokinase, thus preventing a normal glucose sensing and appropriate insulin secretion [121–123]. To test whether the decrease in glucose uptake could be responsible for the impairment of insulin secretion observed in rats treated with a single low-dose injection of TCDD, we measured both GLUT2 protein levels by western blotting and pancreatic glucose uptake by using its nonmetabolizable ^3^H-labeled analog, 2-deoxyglucose. Despite that immunoblotting did not reveal apparent differences in GLUT2 protein levels between pancreatic islets isolated from control and TCDD-treated rats, we showed a significant decrease (−20%) of glucose uptake in the pancreas of TCDD-treated rats [113]. TCDD is thought to affect the levels of glucose transporters, not directly through, e.g., its binding to the glucose transporter protein, but rather by the inhibition of the transcriptional and translational expression of GLUT genes after binding to the cytosolic aryl-hydrocarbon receptor (AhR) [108–110]. However, it has been recently shown that in 3T3-L1 adipocytes, the inhibition of AhR by alpha-naphthoflavone did not prevent the inhibitory effect of TCDD on glucose uptake, suggesting that this effect could be AhR-independent [101]. The history of the search for the action mechanism of TCDD has been largely based on the paradigm of ligand-induced activation of the AhR leading to direct activation of many target genes (the “genomic” or classical pathway) [124–126]. The AhR-mediated biological responses include induction of metabolic enzymes, body weight loss, immunosuppression, skin lesion and changes in cellular growth and differentiation [127]. With particular reference to glucose and lipid metabolism, it may be interesting to note that some biochemical alterations observed in the development of type 2 diabetes (e.g., the inhibition of PEPKC activity, the induction of fatty acid synthesis and the effects on IL-1β and TNF-α) have been also reported in TCDD-treated rats [85,92,128–135] and in TCDD-exposed mouse hepatocytes [136,137]. Some of these effects (expression of key gluconeogenic genes, PEPKC and G6Pase) have been very recently attributed to the TCDD-induced activation of the AhR target gene, TiPARP (TCDD-inducible poly(ADP-ribose)polymerase) [138].

### 3.2. The Non-Genomic Pathway of Dioxin Toxicity

In the last decade, it has been observed that many of the major risk factors for type 2 diabetes (overnutrition, low dietary fiber, sedentary lifestyle, sleep deprivation and depression) can induce local or systemic low-grade inflammation. This inflammatory response is transient or milder in individuals not at risk for type 2 diabetes. By contrast, inflammation in response to lifestyle factors is more relevant and sustained in individuals at risk for type 2 diabetes and appears to occur also in the pancreatic islets (for a recent review, see [2]). The long-term consequences of prolonged low-grade inflammation, in particular in response to metabolic stress, could result in being detrimental [139] and eventually lead to overt diabetes if counter-regulatory circuits to inflammation are compromised as a consequence of a genetic and/or epigenetic predisposition [2,140,141]. In view of the above considerations, it is pertinent to highlight that one of the major actions of TCDD is to elicit inflammatory cellular responses, which can be very intense and last for long time periods [142–145]. In particular, it has been shown that TCDD rapidly induces a significant rise of intracellular [Ca^++^], which, in turn, activates cytosolic phospholipase A_2_ enzyme activity, as demonstrated by the early release of arachidonic acid, followed by activation of Src kinase and induction of several inflammatory markers. This new, inflammation-inducing action pathway of TCDD is definitely distinguished from the classical action pathway on the basis of evidence clearly indicating differences in the timing of activation of the two pathways, the possibility to be selectively blocked by specific inhibitors (e.g., calcium-signaling blockers) without affecting the DRE-driven CYP1A1 induction and the non-involvement of Arnt in the new pathway [146–151]. On the basis of these observations, Matsumura designated the newly delineated pathway as the “non-genomic” inflammation pathway of the action of TCDD [152].

This new intriguing theoretical framework could represent a major breakthrough to study the mechanisms of dioxin toxicity in pancreatic beta cells. As beta cell dysfunction is increasingly recognized to play a fundamental role in type 2 diabetes pathophysiology [153,154], it became particularly relevant to investigate the mechanisms that can disrupt beta cell function [155]. In the last few years, in the attempt to clarify the mechanisms underlying the diabetogenic action of dioxin, we utilized as an experimental model the INS-1 cell line, which was established from an X-ray-induced rat transplantable insulinoma and whose cells retain the ability to dose-dependently secrete insulin when stimulated with increasing concentrations of glucose. The maintenance of this functional property makes these cells a valuable and widely used beta cell model [156,157]. By using this model, we observed that 1 h of exposure to TCDD concentrations between 12.5 and 25 nM induced a sharp decline of cell survival (below 20%) [158]. Such “*in vitro*” concentrations could appear quite elevated when compared with the mean body burden for TCDD and other less toxic related cogeners in the general population (estimated to be around 13 ng TEQ/kg body weight), but it should be considered that in particular conditions (such as after industrial accidents), the body burden can increase to remarkably higher levels (up to 7000 ng TEQ/kg b.w.) [12,159]. Thus, at least in such conditions of high environmental exposure, pancreatic β-cells are likely to undergo relevant damage. On the other hand, for comparison, we recall that well-known β-cytotoxic agents, such as alloxan, can produce similar effects on INS-1 cell viability only upon prolonged exposure to much higher concentrations (0.5 mM for 24 h) [160]. Taking into account the “non-genomic” pathway of dioxin toxicity as proposed by Matsumura [146], the observation that in our model, cytotoxic concentrations of TCDD very rapidly (few seconds) induced a dose-dependent increase in intracellular calcium concentration and that blocking calcium entry by EGTA significantly decreased TCDD cytotoxicity is particularly relevant [158]. The early action of TCDD to cause a rapid increase in the cytosolic concentration of Ca^++^ has been initially demonstrated by Hanneman *et al.* [161] and Puga *et al.* [162]. Other laboratories have confirmed such an early rise in intracellular calcium by TCDD in various cell types and tissues [151,163–172]. The mechanism of the TCDD-induced increase of cytoplasmic calcium concentration has not yet been clarified. A primary route for Ca^2+^ influx is through “store-operated channels” in the cell membrane (originally termed capacitative Ca^2+^ entry), probably activated by a fall in Ca^2+^ within the endoplasmic reticulum [173]. Our data are consistent with such a mechanism: indeed, we showed that the TCDD-induced increase in cellular calcium can be attributed both to a transient and quantitatively modest calcium release from intracellular stores and to a more relevant and sustained influx of extracellular calcium into the cell [158]. Overall, these results clearly indicate that TCDD causes calcium influx in a manner independent from the classic action pathway of DRE-driven transcriptional mechanisms and that, very likely, calcium can be viewed as the initial trigger for this nongenomic action of TCDD. It is well known that perturbation in cellular calcium homeostasis may be associated with the early development of cell injury (for a review, see, e.g., [174]). Therefore, the TCDD-induced increase of intracellular [Ca^2+^] might play an important role in the mechanism of dioxin toxicity in beta cells, and a major target for its negative effects could likely be represented by mitochondria.

### 3.3. Dioxin-Induced Mitochondrial Dysfunction

It is known that by the accumulation of calcium when its cytosolic levels are high, mitochondria can play crucial roles in coordinating the complexities of intracellular calcium-signaling pathways [175]. On the other hand, it is also known that calcium can represent a pathological stimulus, eventually leading to apoptotic cell death. To explain this apparent paradox (Ca^2+^ levels as both a physiological and pathological effector of mitochondrial function), it has been proposed that Ca^2+^ could modulate mitochondrial ROS production by several mechanisms (stimulation of the TCA cycle and oxidative phosphorylation; stimulation of nitric oxide synthase; perturbation of mitochondrial antioxidant status) [175]. Indeed, it has been shown that TCDD can increase mitochondrial ROS production [176], probably through a modification of the mitochondrial GSSG/GSH ratio [177,178]. Thus, to study the effect of TCDD on mitochondrial function, we assayed the mitochondrial membrane potential in INS-1 cells after 10 min of exposure to TCDD, and we observed a significant, dose-related mitochondrial depolarization [158]. In β-cells, mitochondria are crucial for the physiological stimulus-secretion coupling, in which the mitochondrial metabolism of pyruvate, glycolitically derived from glucose, generates ATP, which promotes the closure of ATP-sensitive K^+^ channels and the consequent cell depolarization, inducing Ca^2+^ influx through voltage-gated Ca^2+^ channels, increased cytosolic [Ca^2+^] and finally triggering insulin exocytosis [179]. Thus, it is easy to predict that the TCDD-induced damage of mitochondrial function could result in an impaired secretory capability of beta cells. Indeed, we found that a 1-h exposure of INS-1 cells to very low TCDD concentrations (0.05–1 nM) dramatically impaired glucose-stimulated insulin secretion, showing, for the first time, that dioxin, early and at very low doses, can induce significant beta cell dysfunction [158]. We also demonstrated that in TCDD-treated INS-1 cells, the KCl-stimulated insulin secretion (independent of glucose metabolism) is well preserved, strongly suggesting that mitochondrial alterations rather than the exocytotic process might play a crucial role [158]. In this regard, it is important to note that the inhibitory effect of TCDD on glucose-stimulated insulin secretion was observed with 1 nM TCDD, *i.e.*, well below the cytotoxic concentrations. However, it should be noted that at this concentration, TCDD is still able to cause a small, although not significant, mitochondrial depolarization and that electron microscopy indicated that several cells, with an ultrastructural morphology that was otherwise normal, nevertheless contained mitochondria presenting slight pathological alterations, which result in being significantly different from controls when analyzed by quantitative morphometry [180,181]. Slight alterations of the mitochondrial inner membrane ultrastructure have been described in a number of pathological conditions and have been associated with defects of mitochondrial function [182,183]. Interestingly, we have also shown that dehydroascorbate and epigallocatechin 3-gallate (EGCG), two compounds that were both able to protect beta cells from dioxin toxicity, were also able to significantly prevent the TCDD-induced mitochondrial depolarization and the impairment of glucose-induced insulin secretion in INS-1 cells [180,181]. In this regard, it is interesting to mention that it has been recently reported that EGCG protects insulin-producing cells against pro-inflammatory cytokine-induced injuries through the mitochondrial pathway [184].

Thus, taken together, our results seem to indicate the mitochondrion as one of the most likely targets of dioxin acute toxicity in beta cells. This conclusion could be particularly relevant in view of the recently emerging evidence showing that environmental toxins, including POPs, can affect mitochondrial function and subsequently induce insulin resistance [185–189]. Indeed, mitochondrial dysfunction can affect a range of functions, from metabolic to signaling pathways, that regulate hormone action. When mitochondrial function is perturbed, diseases involving energy utilization easily result: obesity and insulin resistance are prototypes of these diseases [190]. Furthermore, mitochondrial abnormalities are associated with intracellular lipid accumulation, insulin resistance and the pathophysiology of type 2 diabetes [187,191–193].

### 3.4. POP Exposure and Obesity

POPs are lipophilic compounds that accumulate mainly in adipose tissue. It has been proposed that mitochondrial dysfunction could play an important role in chronic low-grade inflammation in adipose tissue, which is believed to represent a crucial mechanism in the development of type 2 diabetes [140,194]. Experimental animal studies suggest that chronic exposure to low-dose POP mixtures similar to the current human background exposure may cause mitochondrial dysfunction [195], probably trough GSH depletion [196], eventually leading to insulin resistance and diabetes. Moreover, POPs acting as endocrine disrupting chemicals could negatively interfere with the carefully coordinated hormonal regulation of adipocyte metabolism, promoting the development of dyslipidemia and diabetes [197–200]. As a final comment, it is important to remember that although obesity represents a strong risk factor for type 2 diabetes, a subset of obese individuals termed metabolically healthy but obese (MHO) are insulin sensitive with a preserved glucose tolerance, have a less systemic low-grade inflammation and visceral adipose tissue accumulation with respect to metabolically abnormal obese (MAO) subjects and are relatively protected from the development of cardiovascular complications. Very interestingly, Gauthier *et al.* [201] recently reported that MHO phenotypes present significantly lower circulating levels of POPs (dioxin- and non-dioxin-like polychlorinated biphenyls) as compared to the MAO phenotype.

### 3.5. Dioxin-Induced Alteration of Signal Transduction Pathways

Although the largely AhR-independent effect on mitochondrial function could play a key role in dioxin toxicity, other mechanisms leading to beta cell dysfunction can be involved. In this regard, it has been proposed that the non-genomic pathway of dioxin toxicity, initially triggered by the rapid increase in the intracellular [Ca^++^], could be subsequently converted in more stable long-term messages by the activation of selected protein kinases [152]. Our experiments, in agreement with previously reported results obtained in other cell types [202–205], confirm that in beta cells, ERK 1/2 and JNK are activated, whereas Akt and p38 are unmodified by TCDD treatment [177]. The observation that EGCG can prevent the TCDD-stimulated selective activation of ERK and JNK is of particular interest in view of the recently reported ability of selected food phytochemicals to reduce the toxic actions of TCDD in U937 macrophages [206] and of flavones and catechins to suppress the TCDD-induced phosphorylation of ERK 1/2 in mouse hepatoma Hepa-1c1c7 cells [207]. The observed inhibition of TCDD-induced MAPK phosphorylation by EGCG could be related to the reported suppression of the dissociation of hsp90 and XAP2 from AhR by the interaction of EGCG with hsp90 [208]. This mechanism is particularly attractive, because it has been reported that in MCF10A cells, the rapid activation by TCDD of c-Src kinase is mediated by the Cdc37-HSP90 complex [209].

### 3.6. Dioxin-Induced Activation of Autophagy

Further information on the mechanisms of dioxin toxicity can be obtained from the ultrastructural analysis of beta cells after TCDD exposure. Indeed, dioxin cytotoxicity is associated with several ultrastructural alterations, such as extensive degranulation, mitochondrial abnormalities and peripheral nuclear condensation [158]. Among the ultrastructural alterations induced by dioxin in beta cells, one of the most characteristic is the diffuse and relevant activation of autophagy [158,181], which has been confirmed by using different experimental techniques (electron microscopy, monodansylcadaverine fluorescence, immunoblotting), in accordance with the internationally acknowledged guidelines for the study of autophagy [210]. TCDD-induced activation of autophagy was recently observed also in a bovine kidney cell line [211]. These observations could acquire particular relevance in view of the recently reported finding that beta cells in human type 2 diabetes have signs of altered autophagy, which may contribute to the loss of beta cell mass [212]. Like many other cellular defense mechanisms, autophagy can be considered as a double-edged sword. On the one hand, it has been recently demonstrated that constitutive autophagy plays important roles in the maintenance of pancreatic beta cell homeostasis [213–216]. On the other hand, autophagy can paradoxically have either pro-survival or pro-death functions, depending on the context [217]. In beta cells, many pieces of evidence indicate that autophagy is a cell survival pathway that can also mediate cell death under certain condition, e.g., when autophagy is overactivated as in response, e.g., to endoplasmic reticulum stress [218] or to particularly severe organelle damage, as that induced by TCDD [181].

## 4. Conclusions

**Figure 1.**
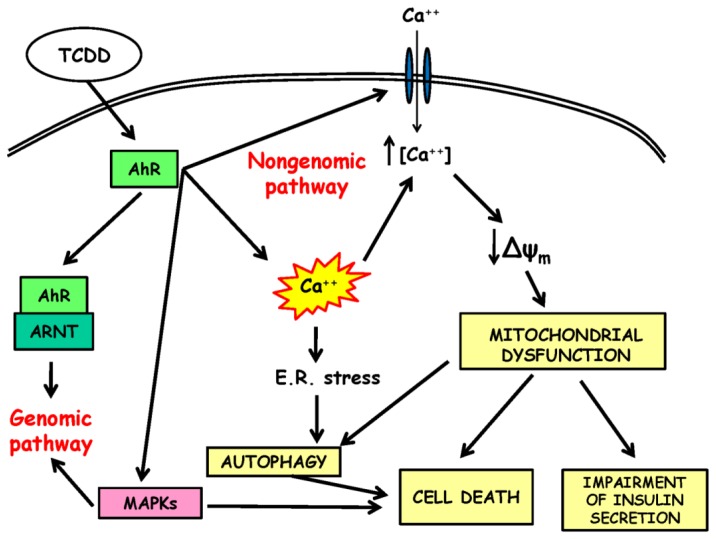
Schematic representation of the proposed pathways involved in the acute dioxin toxicity in pancreatic beta cells. (TCDD, 2,3,7,8-tetrachlorodibenzo-*p*-dioxin; AhR, aryl hydrocarbon receptor; ARNT, aryl hydrocarbon receptor nuclear translocator; MAPKs, mitogen-activated protein kinases).

In the last few decades, a considerable body of epidemiological evidence has been accumulated, whose conclusions strongly suggest that exposure to dioxin and other POPs can be considered as a new risk factor for diabetes in humans in addition to the traditional lifestyle-related factors, such as excess of energy intake and a lack of exercise [14,43]. Consequently, the association between environmental pollutants and diabetes is now regarded as an emerging topic in the field of environmental health sciences and could be rightly considered as one of the “paradoxes of progress” [5]. On the other hand, an increasing number of experimental evidence clearly indicates that pancreatic beta cells can be considered a relevant and sensitive target of dioxin cytotoxicity, throwing some light on the underlying biological mechanisms (Figure 1). The obtained results have turned the attention towards new interesting pathogenetic perspectives, showing the possibility of a new, non-genomic pathway and indicating the mitochondrion as one of the main targets of the acute toxicity of dioxin. However, many basic questions are still to be addressed, and a further experimental effort will be necessary in the years to come to better understand how dioxin exposure could contribute to the worldwide rising prevalence of type 2 diabetes.
